# Early Myocardial Function Affects Endocardial Cushion Development in Zebrafish

**DOI:** 10.1371/journal.pbio.0020129

**Published:** 2004-05-11

**Authors:** Thomas Bartman, Emily C Walsh, Kuo-Kuang Wen, Melissa McKane, Jihui Ren, Jonathan Alexander, Peter A Rubenstein, Didier Y. R Stainier

**Affiliations:** **1**Department of Biochemistry and Biophysics, University of CaliforniaSan Francisco, San Francisco, CaliforniaUnited States of America; **2**Department of Pediatrics, University of CaliforniaSan Francisco, San Francisco, CaliforniaUnited States of America; **3**Department of Pediatrics, University of Cincinnati College of MedicineCincinnati, OhioUnited States of America; **4**Divisions of Neonatology, Pulmonary Biologyand Developmental Biology, Cincinnati Children's Hospital Medical Center, Cincinnati, OhioUnited States of America; **5**Whitehead Institute for Biomedical Research, CambridgeMassachusettsUnited States of America; **6**Department of Biochemistry, University of Iowa College of MedicineIowa City, IowaUnited States of America; **7**Division of Pulmonary and Critical Care Medicine, University of CaliforniaSan Francisco, San Francisco, CaliforniaUnited States of America

## Abstract

Function of the heart begins long before its formation is complete. Analyses in mouse and zebrafish have shown that myocardial function is not required for early steps of organogenesis, such as formation of the heart tube or chamber specification. However, whether myocardial function is required for later steps of cardiac development, such as endocardial cushion (EC) formation, has not been established. Recent technical advances and approaches have provided novel inroads toward the study of organogenesis, allowing us to examine the effects of both genetic and pharmacological perturbations of myocardial function on EC formation in zebrafish. To address whether myocardial function is required for EC formation, we examined s*ilent heart (sih^−/−^)* embryos, which lack a heartbeat due to mutation of *cardiac troponin T* (*tnnt2*), and observed that atrioventricular (AV) ECs do not form. Likewise, we determined that cushion formation is blocked in *cardiofunk (cfk^−/−^)* embryos, which exhibit cardiac dilation and no early blood flow. In order to further analyze the heart defects in *cfk^−/−^* embryos, we positionally cloned *cfk* and show that it encodes a novel sarcomeric actin expressed in the embryonic myocardium. The Cfk^s11^ variant exhibits a change in a universally conserved residue (R177H). We show that in yeast this mutation negatively affects actin polymerization. Because the lack of cushion formation in *sih-* and *cfk-*mutant embryos could be due to reduced myocardial function and/or lack of blood flow, we approached this question pharmacologically and provide evidence that reduction in myocardial function is primarily responsible for the defect in cushion development. Our data demonstrate that early myocardial function is required for later steps of organogenesis and suggest that myocardial function, not endothelial shear stress, is the major epigenetic factor controlling late heart development. Based on these observations, we postulate that defects in cardiac morphogenesis may be secondary to mutations affecting early myocardial function, and that, in humans, mutations affecting embryonic myocardial function may be responsible for structural congenital heart disease.

## Abstract 2

Cardiac anomolies can result from very early defects in heart development. In zebrafish, such defects have been shown to be caused by a new gene called *cardiofunk*

## Introduction

The genetic programs and developmental processes that lead to organ formation are still poorly understood. We are currently witnessing an expansion in research that aims to identify the genes responsible for the structural development of organs and their later function. Among the organs of the body, the heart is unique because it begins to function mechanically before structural development is complete, begging the important question of whether myocardial function is required for the morphogenetic events that occur after the heart begins beating. One of the late steps of heart development is the formation of the endocardial cushions (ECs), which are tissue swellings that develop in characteristic locations along the anterior–posterior (AP) extent of the heart tube and contribute to valves and, in four-chambered hearts, to septae. Because of the clinical significance and prevalence of EC defects in humans (Hoffman 1995), an understanding of the genetic and epigenetic factors controlling cushion and valve formation is critical.

During the process of EC and valve development, specific endocardial cells undergo multiple poorly understood specification, differentiation, and migration events en route to becoming functional heart valves. The genes involved in one substep of this process, epithelial–mesenchymal transformation (EMT), are gradually being identified. Analysis of EMT during cardiac-cushion development has implicated molecules such as Fibronectin, Transferrin, ES-130, hLAMP-1, TGF-β2, TGF-β3, BMP-2 (reviewed in Nakajima et al. 2000), Alk-3 (Gaussin et al. 2002), and hyaluronic acid (Camenisch et al. 2000) as being required for this process. However, many other molecules are likely to be involved in the complex process of EMT, and little is known about the events leading to heart-valve formation that precede or follow EMT, prompting us to take a forward genetic approach to this question (Stainier et al. 2002).

To examine the role of epigenetic factors involved in EC formation, Hove et al. (2003) surgically manipulated zebrafish embryos and put forth the hypothesis that shear stress on endocardial cells is required for EC development. In our study, we use both genetic and pharmacological approaches to test the importance of myocardial function in EC development.

## Results

### *sih* ^−/−^ Embryos Lack ECs

**Figure 1:**
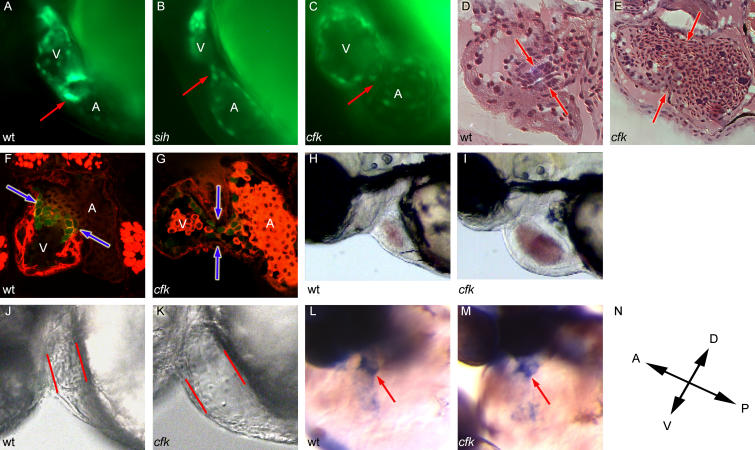
Embryos with Defective Myocardial Function Do Not Form AV ECs (A–C) Fluorescence micrographs of embryos carrying a tie2::GFP transgene, visualized at 48 hpf. In (A), the endocardial ring is visible as a collection of GFP-positive cells at the AV boundary in wild-type (wt) embryos (red arrow). In (B), *sih^−/−^* embryos fail to form an AV ring at 48 hpf. In (C), *cfk^−/−^* embryos fail to form an AV ring at 48 hpf. (D and E) Cushion development remains defective in *cfk^−/−^* embryos. In (D), a 5 μm hematoxylin and eosin-stained plastic section shows the initial stages of cushion development at the AV boundary (red arrows) in a 72 hpf wild-type embryo, with the ECs being two to three cell layers thick at this stage. In (E), a *cfk* ^−/−^ embryo at 72 hpf shows dilation of both chambers of a blood-filled heart with no evidence of cushion formation at the AV boundary (red arrows). (F and G) *cfk^−/−^* embryos fail to form ECs at late stages. Embryos were visualized at identical magnification after counter-staining with rhodamine phalloidin. Red blood cells (RBCs) are seen in the atria of the hearts. In (F), confocal microscopy of a 96 hpf wild-type heart from a tie2::GFP line shows triangular ECs at the AV boundary (blue arrows). In (G), *cfk^−/−^* embryos at 96 hpf lack cushion formation and clustering of GFP-positive cells at the AV boundary (blue arrows). (H) At 72 hpf, wild-type embryos have narrow hearts with forward blood flow through the embryo. (I) At 72 hpf, *cfk^−/−^* embryos have dilated hearts filled with blood that regurgitates freely from the ventricle to the atrium. (J and K) The initial phenotype in *cfk^−/−^* embryos is cardiac dilation at 36 hpf. In (J), wild-type embryos have a narrow ventricle and generate pulsatile flow at 36 hpf. In (K), *cfk^−/−^* embryos have an increased end-diastolic diameter (on average 1.18× wild-type, p < 0.01) and do not generate blood flow at 36 hpf. (L and M) Increased *bmp-4* expression at the AV boundary (red arrow) is observed in wild-type (L) and *cfk^−/−^* (M) embryos at 42 hpf in anticipation of endocardial ring formation. (N) Orientation of the embryos shown in (L) and (M).

In zebrafish, an initial step of EC development is the formation of the endocardial ring, a structure generated by the clustering of endocardial cells at the atrioventricular (AV) boundary. This process is easily visualized at 48 h postfer-tilization (hpf) by examining the endocardial cells expressing green fluorescent protein (GFP) under the control of the mouse *tie2* promoter (Motoike et al. 2000; Walsh and Stainier 2001) (Figure 1A). We have previously reported the early cardiac phenotype of *silent heart (sih^−/−^)* embryos, which establish neither a heartbeat nor blood flow due to mutation of *cardiac troponin T (tnnt2)*, but do undergo looping morphogenesis (Sehnert et al. 2002). We crossed the *sih* mutation into the tie2::GFP line and found that although *sih^−/−^* embryos are otherwise morphologically normal, they fail to form an endocardial ring at the AV boundary (Figure 1B). Because *sih^−/−^* embryos do not have a heartbeat and therefore no blood flow, it is unclear from this observation whether the defect in EC formation is due to lack of myocardial function or lack of shear stress on endocardial cells.

### *cfk* ^−/−^ Embryos Lack ECs Subsequent to Impaired Function

Through genetic screens (Alexander et al. 1998; Stainier et al. 2002) we have identified a new mutant, *cardiofunk (cfk)*, that fails to accumulate tie2::GFP-positive cells at the AV boundary by 48 hpf (Figure 1C). We further examined EC development in *cfk^−/−^* embryos histologically. By 72 hpf, wild-type embryos have developed AV ECs that are more than one cell-layer thick (Figure 1D), while *cfk^−/−^* embryos show no evidence of cushion formation (Figure 1E). Examination by confocal microscopy further shows that wild-type embryos have well-developed cushions by 96 hpf (Figure 1F), whereas *cfk^−/−^* embryos still show no evidence of an endocardial ring or cushion formation (Figure 1G). Thus, EC formation appears to be defective from an early stage, and not simply delayed, in *cfk^−/−^* embryos. The lack of EC development in these mutants leads to toggling of the blood between the atrium and ventricle and its accumulation inside the heart by 48 hpf (Figure 1H and Figure 1I; see also Video S1). Interestingly, the earliest observable phenotype in *cfk^−/−^* embryos is cardiac dilation, as evidenced by an increase in ventricular end-diastolic diameter at 36 hpf (Figure 1J and Figure 1K), at which time *cfk^−/−^* embryos have failed to establish a circulation (Video S2). The cardiac dilation and lack of circulation in *cfk^−/−^* embryos are nearly fully penetrant phenotypes, while the failure of EC development occurs in about 50% of *cfk^−/−^* embryos. This observation raises the possibility that the lack of EC formation may result secondarily from some other defect, such as changes in myocardial function or shear stress on endocardial cells.

### AV Boundary Specification Is Not Affected in *sih* ^−/−^ and *cfk* ^−/−^ Embryos

To determine more precisely which step of EC formation is affected in *sih^−/−^* and *cfk* ^−/−^ embryos, we examined the expression of *bmp-4*, a gene implicated in EC morphogenesis (Eisenberg and Markwald 1995). In wild-type embryos, *bmp-4* is initially expressed throughout the anterior–posterior (AP) extent of the heart tube before becoming localized to the AV boundary at 42 hpf (Walsh and Stainier 2001) (Figure 1L). In *sih^−/−^* and *cfk^−/−^* embryos, *bmp-4* expression similarly becomes restricted to the AV boundary by 42 hpf (Figure 1M) (data not shown)*.* Therefore, the lack of endocardial ring formation in *sih^−/−^* and *cfk^−/−^* embryos does not appear to be due to a defect in specification of the AV boundary, as assessed by *bmp-4* expression, nor general arrest of cardiac development. The wild-typelike restriction of *bmp-4* expression in these mutant embryos contrasts with the situation in *jekyll^−/−^* embryos, which also lack ECs, but in which *bmp-4* expression does not become restricted to the AV boundary (Walsh and Stainier 2001).

### *cfk* Encodes a Sarcomeric Actin

**Figure 2:**
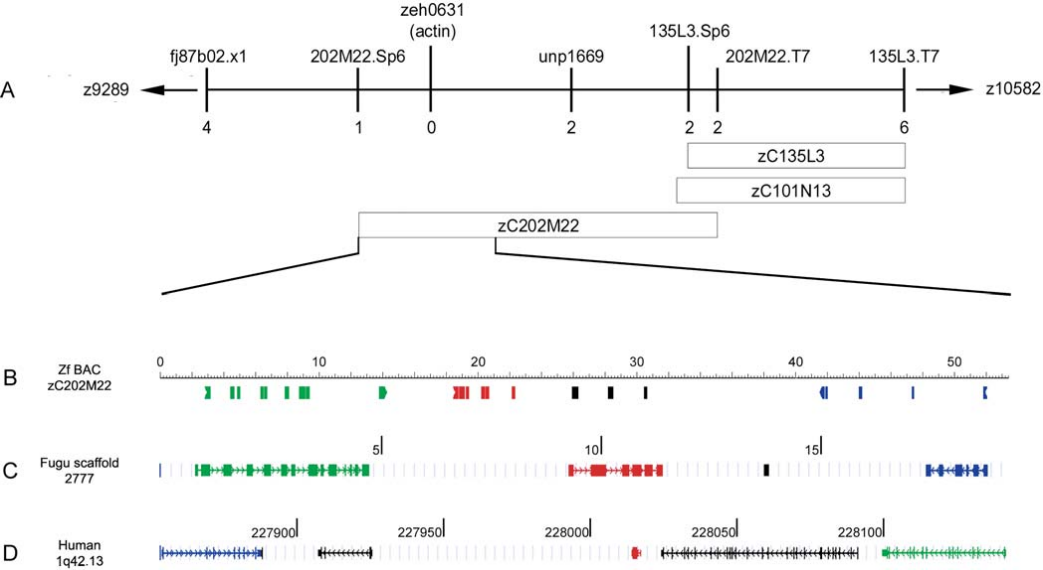
Positional Cloning of *cfk* (A) The locus on zebrafish LG13 containing *cfk* is shown, with the number of recombinants indicated below each marker. The one recombinant at the Sp6 end of BAC zC202M22 and the two recombinants at the T7 end of the same BAC define the critical region. (B) The first 52 kb of zebrafish BAC zC202M22 is shown, including the Sp6 end. One of the two recombinants from the T7 end of the BAC is still present at a marker at 52 kb, narrowing the critical region to this span. GenScan and BLAST analyses identified four coding sequences in this span, including *abc-b10* (green), actin (red), a novel EST *fv49b10* (black), and *rab4a* (blue). (C) Each of the three known genes identified on BAC zC202M22 has a homologue on scaffold 2777 of Fugu rubripes, in the same orientation (green, *abc-b10*; red, actin; blue, *rab4a*). (D) The same three genes lie in proximity to each other and in the same order on human Chromosome 1q42.13 (green, *abc-b10*; red, actin; blue, *rab4a*). Units in black are genes, predicted genes, or ESTs, which are unique in this region to that particular organism.

To understand better the molecular basis for the *cfk* phenotypes, we isolated *cfk* by positional cloning. *cfk* was initially localized to a region of LG13 by bulk-segregant analysis; fine-mapping placed *cfk* between Z9289 and Z10582 (Shimoda et al. 1999). Analysis of 2,034 meioses allowed us to perform a chromosomal walk that restricted *cfk* to bacterial artificial chromosome (BAC) zC202M22 (Figure 2A). Following the sequencing of zC202M22, a portion of the BAC insert was assembled as a 52 kb contiguous sequence, and *cfk* was genetically localized to this region. Using GenScan (Burge and Karlin 1997) and BLAST analysis, we identified four genes in this 52 kb region: *abc-b10*, *rab4a*, actin (*zeh0631*), and *fv49b10* (Figure 2B). Comparison of this region of the BAC to fugu and human sequences identified regions of conserved synteny on fugu scaffold 2777 (Figure 2C) and human Chromosome 1q42.13 (Figure 2D) and confirmed our GenScan analysis. The four zebrafish genes in the critical interval were analyzed by sequencing from *cfk^−/−^* mutants and reverse genetic techniques using morpholino antisense oligonucleotides. Injections of morpholinos against each of the four coding sequences showed no obvious cardiac phenotype, indicating that we might not be working with a loss-of-function mutation or that *cfk* has overlapping function with another gene. Subsequently, sequencing of the actin gene (*zeh0631*) showed a change of arginine 177 to histidine. The three embryos recombinant at either end of the 52 kb region were homozygous at the site of the R177H lesion, suggesting that we had isolated *cfk*.

**Figure 3:**
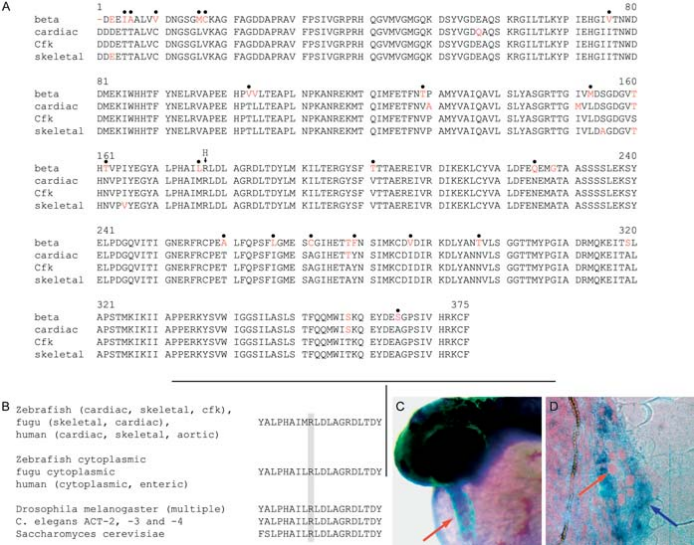
Sequence and Expression Analysis of *cfk* (A) *cfk* encodes a sarcomeric actin highly homologous to zebrafish α-cardiac and α-skeletal actins. Cfk differs from zebrafish α-cardiac actin at six residues and from zebrafish α-skeletal actin at four residues, but from zebrafish β-actin at 28 residues. Residues in red are those that differ from Cfk. Dots above the sequence indicate residues that universally distinguish sarcomeric from cytoplasmic actins. The arrow at R177 indicates the location of histidine in Cfk^s11^. (B) The arginine at position 177 is universally conserved in all actin proteins examined. (C and D) *cfk* is expressed in the myocardium during development. In (C), whole-mount in situ hybridization on a cmlc2::GFP embryo at 36 hpf shows that *cfk* is expressed throughout the AP extent of the heart tube. Blue staining indicates areas of *cfk* expression; green is the region of cmlc2 expression. The red arrow indicates the heart tube. In (D), a plastic section of stained embryo shows *cfk* expression in the myocardium of the heart (blue arrow), but not in the endocardial cells (red arrow). From the onset of its expression in the heart region (around the 16-somite stage), *cfk* does not appear to be expressed in endothelial and endocardial cells. Weak *cfk* expression is also seen in the somites (data not shown).

The cardiac dilation caused by the *cfk* mutation supported our hypothesis that *cfk* corresponded to an actin gene. Analyses of Cfk indicate that it is a sarcomeric actin by virtue of its homology to zebrafish α-cardiac and α-skeletal actins and lack of homology to zebrafish cytoplasmic actin, as well as its conservation of synteny with the human skeletal actin. Further evidence that *cfk* encodes a sarcomeric actin and not a cytoplasmic actin includes the presence of an extra residue at the N-terminus of Cfk, which is seen in all sarcomeric actins but no cytoplasmic actins, as well as the presence of residues that are stereotypic for sarcomeric actins at all 20 locations where sarcomeric and cytoplasmic actins have unique amino acids (Khaitlina 2001) (Figure 3A). Searches of GenBank databases identified both zebrafish α-cardiac– and α-skeletal–actin genes that are well represented in the zebrafish expressed sequence tag (EST) collection and are related to, but clearly distinct from, *cfk* (see Figure 3A). Analysis of multiple actins from several species revealed that the R177 residue, which is mutated to histidine in the *cfk^s11^* allele, is universally conserved (Figure 3B). In vitro mutations at the R177 residue usually affect actin polymerization or function, although the biochemical effect of an R117H transition has not been tested.

To determine the expression pattern of *cfk*, we performed in situ hybridization with a probe corresponding to its 3′ untranslated region (UTR), which is distinct from the 3′ UTR sequences of zebrafish α-cardiac and α-skeletal actins. These data showed that *cfk* is expressed in the myocardial but not endocardial cells of zebrafish embryos from 24 to 48 hpf (Figure 3C and Figure 3D), suggesting a nonautonomous role in EC formation. Taken together, the tight linkage between *cfk* and this actin gene, the presence of a significant genetic lesion in the actin gene, and the expression profile of the actin gene indicate that *cfk* corresponds to the actin gene previously identified by the EST *zeh0631*.

### The R177H Mutation Alters Actin Polymerization

**Figure 4:**
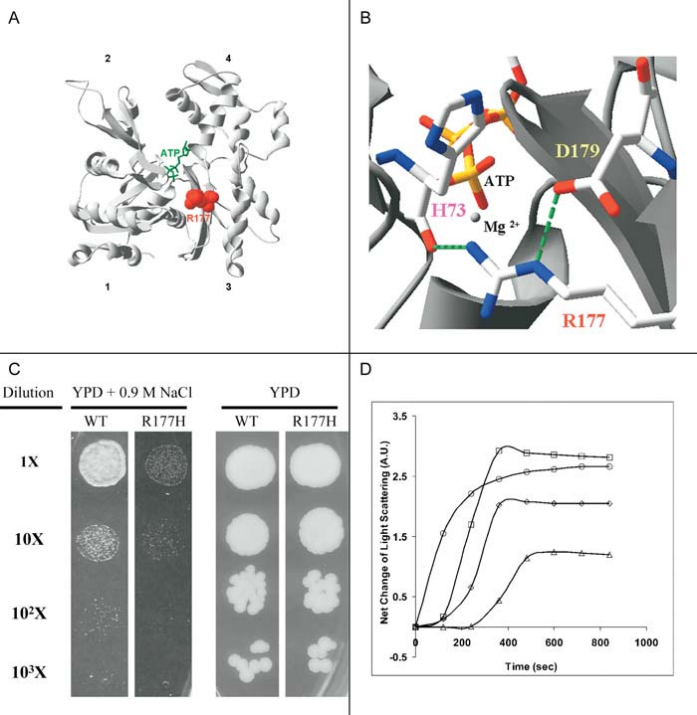
An Arginine to Histidine Change at Position 177 of Cfk Alters the Location of Positive Charges in the Nucleotide Binding Cleft of Actin (A) The position of R177 (red) in the structure of the yeast-actin monomer. The bound ATP is shown in green. The structure shown is based on a crystal structure of actin (PDB:1YAG). (B) Magnification of the cleft region showing the hydrogen bonding (green dashed lines) involving R177, which will be disrupted in H177. (C) The R177H mutation restricts yeast growth under hyperosmolar stress. Wild-type (wt) and mutant cells were grown to a density of 3 × 10^6^ per milliliter, and aliquots were plated either on YPD or YPD plus 0.9 M NaCl at different dilutions of the culture. The cells were then incubated at 30°C for 72 h. The normosmolar and hyperosmolar experiments were done at different times. (D) Purified R177H actin has a higher critical concentration of polymerization and a delayed nucleation phase. Wild-type or mutant actin was purified from yeast cultures and polymerization mea-sured by light diffraction. Symbols and abbreviations: circles, 5 μM wild-type actin; triangles, 5 μM R177H actin; diamonds, 7.5 μM R177H actin; squares, 10 μM R177H actin; ATP, adenine triphosphate; wt, wild-type.

Previous biochemical studies have revealed a critical role for R177 in actin polymerization. An R177A yeast variant is heat sensitive and does not grow in 0.9 M NaCl (Wertman et al. 1992; Drubin et al. 1993), while an R177D mutation in chick β-actin leads to severely altered polymerization properties in vitro (Schuler et al. 2000). These results suggest that a positive charge at position 177 is important for actin function in vivo (Wriggers and Schulten 1999). The R177H *cfk^s11^* mutation represents a less severe change in charge than the previously mentioned yeast or chick mutations. To assess the effect of the R177H mutation on actin function, we used site-directed mutagenesis to generate a haploid yeast strain in which the R177H mutant actin was the only actin expressed in the cell. These cells were readily obtained and showed no significant altered morphology or growth characteristics on normosmolar complete medium at 30°C. However, the R177H mutation produced a severe growth defect, similar to the R177A mutation, when the cells were incubated in hyperosmolar complete medium containing 0.9 M NaCl (Figure 4C).

To determine whether the R177H mutation affected actin polymerization, we purified the actin from R177H cells (Cook et al. 1991) and assessed the extent of polymerization by the increase in light scattering as a function of time. The mutant actin exhibited two distinct differences in comparison to a similarly prepared sample of wild-type yeast actin (Figure 4D). First, the extent of polymerization of a quantity of actin equal to that of the wild-type sample was significantly decreased as judged by the final plateau. Second, there was a prolonged apparent nucleation phase not seen with wild-type actin, even at concentrations of mutant actin that produced more F-actin than the wild-type sample. Samples of the polymerization solution were negatively stained with uranyl acetate and examined by electron microscopy to confirm that the increase in light scattering was caused by F-actin formation and not merely aggregation (data not shown). To define better the apparent difference between R177H and wild-type actins in regard to the critical concentration necessary for polymerization, we assessed the extent of polymerization of different amounts of actin relative to the amount of total actin using a light-scattering assay. Based on determinations with two independent preparations of actin, the critical concentration is 0.3 μM for wild-type actin and 1.9 μM for the mutant actin, confirming that this apparently mild mutation exerts a drastic effect on actin-filament stability.

**Figure 5:**
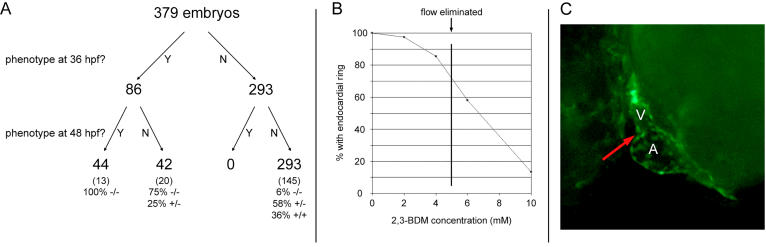
Lack of EC Formation Is Secondary to Defective Myocardial Function (A) Embryos were observed for defective myocardial function and lack of blood flow at 36 hpf and lack of endocardial ring formation at 48 hpf. Approximately half of the embryos with defective myocardial function at 36 hpf did not develop endocardial rings. All embryos with a lack of endocardial rings previously demonstrated a myocardial function phenotype at 36 hpf. Further analyses including the genotyping of a subset of embryos showed that a small percentage of *cfk^−/−^* embryos were unaffected at 36 hpf and that they subsequently developed ECs. (B) Embryos treated from 24 to 48 hpf with 2,3-BDM to decrease myocardial force failed to develop endocardial rings in a dose-dependent manner. Loss of ring formation was not linked to loss of blood flow—14% of embryos treated at 4 mM 2,3-BDM did not form rings despite the presence of blood flow, and 58% of embryos treated at 6 mM did form rings despite the absence of blood flow. (C) Example of an embryo treated with 10 mM 2,3-BDM that failed to develop an endocardial ring at the AV boundary (red arrow).

During our work with *cfk^s11^*, we observed that some embryos showing the *cfk* phenotype were *cfk^+/−^* (Figure 5A), suggesting that the R177H mutation can exert a partially dominant effect. Indeed, subsequent experiments (Wen and Rubenstein 2003) demonstrate that the presence of the mutant actin in a solution of wild-type actin exerts a partially dominant effect on actin polymerization. The partial dominance of *cfk^s11^* and the yeast data support a model whereby substantial copolymerization of Cfk^s11^ actin monomers with wild-type monomers (either Cfk or α-cardiac actin) leads to unstable filaments, occasionally giving rise to a phenotype in heterozygous embryos.

### *cfk* Affects EC Development through Its Effect on Myocardial Function

Because most *cfk^−/−^* embryos have a dilated heart and lack blood flow and because subsequently approximately 50% of these embryos fail to form ECs, we wondered whether these two phenotypes were causally related or whether lack of EC formation could occur independently of poor early myocardial function. To address this question, 379 embryos from multiple *cfk* clutches were assayed for cardiac dilation and lack of blood flow at 36 hpf and for lack of endocardial ring formation at 48 hpf. Embryos that were phenotypically wild-type at 36 hpf invariably developed endocardial rings over the next 12 h, whereas all embryos that failed to form endocardial rings had previously shown a functional phenotype at 36 hpf (see Figure 5A). Importantly, the few *cfk^−/−^* embryos that were phenotypically wild-type at 36 hpf remained so at 48 hpf. These data strongly suggest that lack of EC formation occurs secondarily to poor myocardial function or lack of blood flow at 36 hpf.

### Myocardial Function, Not Shear Stress, Is Likely Required for EC Formation

Because *cfk^−/−^* and *sih^−/−^* embryos each exhibit both a myocardial phenotype (dilation and silence, respectively) and fail to generate blood flow, it is impossible to conclusively state whether EC formation is affected in these embryos directly as a result of poor myocardial function or indirectly as a result of the perturbation in blood flow and shear stress caused by poor myocardial function. Hove et al. (2003) attempted to address a similar question by inhibiting blood flow mechanically without affecting myocardial function. However, other aspects of cardiogenesis were disturbed in their experiments, leaving open the question of which effects of their manipulations were primary and which were secondary. We chose an alternate approach to analyze the respective roles of myocardial function and blood flow in EC development by finding doses of an inhibitor of myofibril function that would or would not affect blood flow. We treated tie2::GFP embryos with various concentrations of 2,3-butanedione monoxime (2,3-BDM), which blocks myofibrillar ATPase in a dose-dependent manner (Herrmann et al. 1992) and decreases myocardial force. As the treatment concentration of 2,3-BDM increased, the percentage of embryos that formed endocardial rings at 48 hpf decreased (Figure 5B). Importantly, blood flow was abolished in all embryos treated with 2,3-BDM at 6 mM or higher, yet 58% of them (*n* = 74) formed an endocardial ring, indicating that blood flow is not required for the initial steps of cushion formation. When myofibril function was further decreased by treatment with 10 mM 2,3-BDM, the percentage of embryos with an endocardial ring decreased to 13% (*n* = 68). Studies with the anesthetic tricaine confirmed the observation that ECs may form in the absence of blood flow and that the likelihood of forming cushions is inversely proportional to the concentration of tricaine (data not shown). In summary, the results from the 2,3-BDM and tricaine treatments suggest that it is poor myocardial function, and not lack of blood flow, which is primarily responsible for the loss of EC formation in *cfk^−/−^* and *sih^−/−^* embryos.

## Discussion

The hearts of *sih^−/−^* embryos fail to beat yet undergo looping morphogenesis and AV boundary specification. The specific absence of EC formation in these embryos clearly demonstrates that myocardial function is required for EC formation. Precisely how myocardial function is required for EC formation is unclear. Prior work has demonstrated a requirement for AV boundary myocardium in the formation of ECs, and several signaling molecules emanating from the myocardium at the AV boundary have been identified (reviewed in Eisenberg and Markwald 1995). Although we have shown that the expression of *bmp-4* is not affected by myocardial function defects, it is possible that myocardial function is somehow required for another aspect of this signaling event.

Hove et al. (2003) recently argued for the importance of intracardiac hemodynamics as a key epigenetic factor affecting embryonic cardiogenesis. In their experiments, blood flow into the heart was eliminated by surgical placement of a bead at the inflow tract. One of the resulting phenotypes in these embryos was lack of EC formation, leading them to hypothesize that a reduction in shear stress on endocardial cells caused this phenotype. However, it is possible that the lack of EC formation observed by Hove et al. (2003) in surgically manipulated embryos was secondary to either the lack of looping observed in these same embryos, an effect on myocardial function by the surgical manipulations, or a more general arrest of cardiac development. In contrast, *sih* ^−/−^ embryos undergo looping morphogenesis, making them perhaps a more appropriate model to analyze, and these embryos also lack ECs. Therefore, myocardial function and/or blood flow appear to play a role in EC development. Data from 2,3-BDM and tricaine treatments, in which many embryos without blood flow still formed ECs, suggest that endocardial shear stress may not be key to EC morphogenesis.

In mouse, a number of studies indicate that mutation of a single gene, including *tbx5* (Bruneau et al. 2001), *nkx2.5* (Schott et al. 1998; Biben et al. 2000), and *has2* (Camenisch et al. 2000), affects both myocardial function and cardiac morphogenesis. These mutations have been thought to affect cardiac structural development and function in parallel pathways or in a causal pathway with the abnormal structure of the hearts leading to abnormal function. Based on our analyses of *cfk,* we would like to propose an alternative interpretation, namely that some of these mutations may primarily perturb early myocardial function, which then disrupts subsequent steps of heart morphogenesis. This model is supported by studies of the *ncx1* null mouse, in which the heartbeat is eliminated and the EMT associated with EC development appears to be defective (Koushik et al. 2001). The *ncx1*studies, together with ours, indicates that EC development may be particularly sensitive to perturbations in myocardial function. However, because of the difficulty in completely unlinking heart function and blood flow, further studies will be required to elucidate the exact contributions of myocardial function and endothelial shear stress on cushion and valve morphogenesis.

Our data show that mutations in two different sarcomeric genes lead to EC defects in zebrafish. Similar mutations affecting myocardial function may thus cause EC defects in humans. While most infants born with EC defects exhibit relatively normal myocardial function, actin-gene expression is known to undergo significant changes during development (Cox and Buckingham 1992). Thus, a mutation in an actin gene expressed in the heart during embryogenesis, when control of actin-gene expression appears to be less specific than in the mature animal, could lead to an EC phenotype without apparent effects on myocardial function at birth. As shown by the *cfk* mutant, it is possible that genetic lesions in actin genes expressed only transiently in myocardial cells could cause embryonic cardiovascular phenotypes, particularly if those lesions act in a dominant manner. Therefore, as we seek to identify the genes responsible for human cardiac malformations, those primarily involved in myocardial function should also be considered.

## Materials and Methods

### 

#### Zebrafish

Zebrafish were maintained and staged as described (Westerfield 2000). We used the *sih^tc300b^* allele (Chen et al. 1996) and the *cfk^s11^* allele identified in a screen in our laboratory (Alexander et al. 1998). Animal protocols were approved by the Committee on Animal Research of the University of California, San Francisco (San Francisco, California, United States).

#### In situ hybridization

We carried out whole-mount in situ hybridization as described elsewhere (Alexander et al. 1998). We used antisense RNA probes to *bmp-4* (Nikaido et al. 1997) and the 3′ UTR of *cfk*. Visualization of GFP-positive cells after in situ hybridization was performed by treating embryos with a rabbit anti-GFP-IgG followed by a fluoresceinated mouse anti-rabbit-IgG.

#### Morpholino antisense “knock-down.”

We designed morpholino oligonucleotides (Gene Tools, Philomath, Oregon, United States) to bind to the initiation codon and flanking sequences of the following zebrafish genes: *sih* (Sehnert et al. 2002), *rab4a* (5′-GTCTCTGACATGACTGACGCTGCGT-3′), *abc-b10* (5′-TCATTCGCAACATTGTCCCATACAT-3′), *fv49b10.y1* (5′-CCGACCTAATTCGCTTGGT-CACCAT-3′), and *cfk* (5′-CATCTTGATGTATTCTTTCTCTGCT-3′). The morpholinos were injected at 4 ng and 8 ng into tie2::GFP embryos at the one-cell stage and examined at 24 to 72 hpf for myocardial function and EC phenotypes. Morpholinos against *abc-b10*, *rab4a*, and *fv49b10.y1* did not affect myocardial function or EC formation. The *cfk* morpholino caused no phenotype, an expected result given the ability of actin genes to compensate for each other when down-regulated (Kumar et al. 1997; Crawford et al. 2002) and the redundancy of *cfk* with α-cardiac actin.

#### Genetic mapping

We genotyped diploid mutant embryos from a *cfk^s11^*-AB/SJD hybrid strain using SSLP with various CA repeat markers and SSCP with various ESTs. SSCP was performed by denaturing PCR products at 95°C for 10 min in 0.7 mM EDTA and 36 mM NaOH and placing on ice before loading on nondenaturing acrylamide gels. BAC library filters for the CHORI-211 (zC) library were obtained from P. de Jong at BACPAC (Oakland, California, United States). Linkage of the R177H mutation to the *cfk^s11^* allele (through genotyping of mutant embryos) was accomplished by performing PCR across the mutation (forward primer = 5′-ATCGTGCTGGACTCTGGTG-3′ and reverse primer = 5′-GAAAGAATAACCGCGCTCAG-3′) and digesting with NsiI, which cuts only the H177 allele. Synteny analysis was performed through http://genome.jgi-psf.org/fugu3/fugu3.home.html (fugu) and http://genome.ucsc.edu (human).

#### Mutation detection

We used a pool of 25 *cfk^s11^* mutant embryos to extract mRNA (Trizol, GIBCO–BRL, Gaithersburg, Maryland, United States) and synthesize cDNA (SuperScript First Strand, Invitrogen, Carlsbad, California, United States). PCR was then performed from the 5′ UTR to 3′ UTR and multiple clones sequenced. To confirm that the amino acid change we saw was a mutation and not a polymorphism in the strain used for mutagenesis, we examined the DNA of four F1 females from the screen (Alexander et al. 1998) in which *cfk* was identified, one of which led to the *cfk* line and thus should be mosaic for the mutation. This female, but not her three sisters, was indeed mosaic for the mutation (data not shown).

#### Pharmacological treatment of embryos

Clutches of dechorionated embryos were placed in 2,3-BDM (Sigma B-0753, Sigma–Aldrich, St. Louis, Missouri, United States) at 24 hpf at concentrations between 2 and 20 mM. This concentration range has been shown to affect myofibrillar ATPase in a dose-dependent manner, with 25% of activity remaining at 10 mM (Herrmann et al. 1992). 2,3-BDM had a rapid onset of action—at 2 and 4 mM, the embryos had visibly weakened heartbeats within minutes, and at 6 mM or greater the myocardial force was weakened enough to eliminate blood flow almost immediately. During the next 24 h, the embryos were examined periodically to ensure that the pharmacological effect remained constant over time. At 48 hpf the presence or absence of the endocardial ring was assayed. For tricaine treatment, dechorionated embryos were placed in a solution of 0.4 mg/ml ethyl 3-aminobenzoate methanesulfonate salt (Sigma A-5040, MS-222, 886–86-2) from 24 to 48 hpf and assayed for myocardial function during that time and for EC formation at 48 to 54 hpf.

#### Yeast-actin biochemistry

Site-directed mutagenesis of the yeast-actin coding sequence in a centromeric plasmid and construction of haploid cells producing only the mutant actin were carried out as described previously (Cook et al. 1992). Wild-type and mutant actins were purified using a DNase I agarose/DEAE-cellulose-based procedure as described previously (Cook et al. 1991). G-actin was stored at 4°C in G-buffer (10 mM Tris–HCl [pH 7.5], containing 0.2 mM ATP, 0.2 mM CaCl_2_, and 0.1 mM DTT) and used within 2 d. Actin polymerization was induced by the addition of MgCl_2_ and KCl to final concentrations of 2 mM and 50 mM, respectively, and polymerization was assessed by the increase in light scattering as a function of time at 25°C in a 120 μl volume in a thermostatted cuvette using a SPEX Fluorolog 3 fluorimeter with excitation and emission wavelengths set at 360 nm.

## Supporting Information

Video S1 Phenotype of Wild-Type and *cfk^−/−^* Embryos at 72 hpf Wild-type hearts propel blood through the vasculature, whereas *cfk^−/−^* hearts have complete regurgitation of blood from the ventricle to the atrium and therefore no forward blood flow.
(2.91 MB MOV).

Video S2 Phenotype of Wild-Type and *cfk^−/−^* Embryos at 36 hpf Wild-type embryos have a narrow, strongly contracting heart and generate effective blood flow. *cfk^−/−^* embryos (two different embryos shown) have dilated hearts with weak contractions and are not capable of generating circulation.
(2.9 MB MOV).

### Accession Numbers

The GenBank (http://www.ncbi.nlm.nih.gov/Genbank/index.html) accession numbers discussed in this paper are for *cfk* (AY222742), zebrafish α-cardiac actin (AF116824), zebrafish α-skeletal actin (AF180887), and zebrafish cytoplasmic actin (AF057040).

## Abbreviations

AP: anterior–posterior
AV: atrioventricular
BAC: bacterial artificial chromosome
2,3-BDM: 2,3-butanedione monoxime
*cfk*: *cardiofunk;* EC
EMT: epithelial–mesenchymal transformation
EST: expressed sequence tag
GFP: green fluorescent protein
hpf: hours postfertilization
LG: linkage group
*sih*: silent heart
tnnt2: *cardiac troponin T;* UTR
